# Nutritional rickets among children admitted with severe pneumonia at Mulago hospital, Uganda: a cross-sectional study

**DOI:** 10.1186/s12887-018-1310-9

**Published:** 2018-10-29

**Authors:** Thereza Piloya, Beatrice Odongkara, Edward Maloba Were, Faith Ameda, Edison Mworozi, Paul Laigong

**Affiliations:** 1 Makerere University, College of Health Sciences, P.O. Box 7072, Kampala, Uganda; 2 Gulu University, Gulu, Uganda; 3 Paediatric AIDS Elizabeth Glazer, Mbarara, Uganda; 4 Mulago, National Referral and Teaching Hospital, Kampala, Uganda; 5 University of Nairobi, Nairobi, Kenya

**Keywords:** Rickets, Pneumonia, Children, Uganda

## Abstract

**Background:**

There’s abundant sunshine in the tropics but severe rickets is still observed. Nutritional rickets is associated with an increased risk of acute lower respiratory infections. Pneumonia is the leading cause of death in the under 5 -year old children with the highest burden in developing countries. Both Pneumonia and rickets are common in the developing countries and may affect clinical presentation and outcome. This study aimed to determine the prevalence and associated factors of nutritional rickets in children admitted with severe pneumonia.

**Methods:**

This was a cross-sectional study of children aged 2–59 months presenting with severe pneumonia at an emergency unit. We enrolled 221 children between February and June 2012 after consent. A pre-coded questionnaire was used to collect data on socio-demographic, nutritional and past medical history. Physical exam was done for signs of rickets and anthropometric measurements. Serum calcium, phosphorus, and alkaline phosphatase (ALP) were assessed. Children with any physical signs of rickets or biochemical rickets (ALP > 400 IU); had a wrist x-ray done. Nutritional rickets was defined as the presence of radiological changes of cupping or fraying and/ or metaphyseal thickening. Severe pneumonia was defined using the WHO criteria.

Statistical analysis was performed using the Stata 10 statistical package. *P*- value < 0.05 was significant.

**Results:**

The prevalence of nutritional rickets among children with severe pneumonia is 9.5%. However, 14.5% had raised ALP (biochemical rickets). The factors independently associated with rickets was an elevated alkaline phosphatase; *p*-value < 0.001, or 32.95 95% CI (10.54–102.93). Other factors like breastfeeding, big family size, birth order were not significantly associated with rickets. Low serum calcium was detected in 22 (9.9%) of the 221 participants. Overall few children with rickets had typical clinical features of rickets on physical examination.

**Conclusion:**

Rickets is a common problem in our setting despite ample sunshine.

Clinicians should actively assess children for rickets in this setting and screen for rickets in those children at high risk even without clinical features.

## Background

Childhood pneumonia continues to be a significant global health problem.

Its the leading cause of morbidity and mortality among children aged less than 5 yrs. [1].

The vast majority of pneumonia-related deaths in children affect the poor in developing countries who are exposed to higher risk factors for developing Acute Lower Respiratory Tract Infections (ALRIs).

Rickets is the commonest presentation of vitamin D deficiency in children [2]. The sun is the major source of vitamin D. Despite ample sunshine in our setting, rickets is common among children presenting to the hospitals.

Clinical rickets has been reported in hospital-based studies to be strongly associated with severe/very severe pneumonia [3–5]. A hospital study from Egypt showed that acute respiratory infections were present in 81% of children with rickets, compared with 58% of controls [6].

A case-control study in Ethiopia found 42% of hospitalised pneumonia cases had rickets, compared to 4% of children admitted for other reasons [7].

In addition to vitamin D deficiency, low calcium intake has been implicated as a cause of rickets in the areas with ample sunlight. Studies in South Africa found that children who presented with active rickets had diets devoid of dairy products and high in grain and vegetables, were older children 4-16 yrs., spent long hours in the sun and had normal serum 25 hydroxyvitamin D (25(OH)D) concentrations and elevated 1,25(OH)2D concentrations [8]. In Ugandan setting where breastfeeding mothers and chidren are not supplemented with vitamin D and the diets of children are predominantly grain based, children may be predisposed to nutritional rickets caused by either vitamin D deficiency or calcium-deficient rickets.

Uganda has a high under-five mortality rate of 64 deaths per 1000 live births [9]. Pneumonia is ranked as one of the leading causes of death in these children. Increasingly, many children are being diagnosed with severe rickets in sunshine abundant areas [10]. However, vitamin D deficiency is not routinely assessed clinically and biochemically even in those who are at high risk in our setting. Furthermore, there is no Vitamin D supplementation or food fortification programme in Uganda for those who are at high risk of deficiency for both vitamin D and calcium. This study serves to generate data to develop further research in this field. In addition, the study will provide a basis to improve clinical assessment of children for rickets and improve screening and management of rickets in a setting with low suspicion for rickets. Vitamin D and calcium supplementation for children at risk for rickets is cheap, easy and a safe intervention.

## Methods

### Study design & setting

This was a cross-sectional study of 221 children admitted with severe pneumonia at the Emergency Acute Care unit of Mulago hospital, Kampala in the period of February to June 2012. Mulago hospital is the National referral and teaching hospital for Makerere University in Uganda. The hospital admits 8–10 children with severe pneumonia daily. Mulago hospital is located in Kampala in the central region of Uganda, majority of the patients seen in the hospital come from suburbs in and around Kampala. It is found at Latitude 0° N at 3865 ft above sea level. Uganda is sunny most of the year with average annual temperature of about 26 degrees Celsius. The rainy season is from March till May and October till November. The study was conducted at the onset of wet season from February to June 2012.

Mulago hospital is surrounded by 5 large slum/informal settlements with informal housing characterised by overcrowding and limited space for yards for children to safely play. Majority of the children attending the emergency unit in Mulago hospital come from these settlements.

The typical clothing of Ugandan children in wet season is overdressing with sweaters and hats especially for children ages < 6 months because of cultural beliefs. However, as the children get older than 1 year there is less covering; no hats but the clothes do not expose too much of the skin of the arms and legs in Kampala although it may vary in different regions of the country.

### Study participants

Participants were children ages 2–60 months admitted with severe Pneumonia (WHO criteria) during the study period whose parents provided written informed consent. We excluded all children with chronic renal failure, hepatic problem, cerebral palsy, chronic gastrointestinal problems and HIV infected children. Children on anticonvulsants and those with familial or vitamin D dependent/resistant rickets were also excluded. Approval to carry out this study was obtained from the School of Medicine Research and Ethics Committee College of Health Sciences, Makerere University.

#### Study procedure

The study team worked on all week days Monday to Friday, 8 am to 5 pm because of availability of laboratory services. All Children presenting to the emergency unit with difficulty in breathing were screened for eligibility by the research assistant. All the eligible children who were unstable at arrival were first stabilised before recruitment into the study. Eligible children were recruited consecutively until the required sample size was achieved. All eligible children had a detailed clinical assessment done including history and clinical examination. Severe pneumonia was diagnosed on a clinical basis according to the World Health Organization criteria [11]. According to WHO protocol, children with history of cough, respiratory distress and on examination having tachypnea i.e. respiratory rate > 50/min for 2 months to 12 months, > 40/min for 12 months to 5 years and chest indrawing, with or without fever (temp > 37.5 °C) or crepitations were taken as having severe pneumonia.

The clinical history included sociodemographics of both participant and the mother, history of sun exposure, outdoor clothing habits of mother/caretaker mode of feeding, dietary history, family size, the rank of the child in the family and monthly income. Other history taken included past medical history, and growth and developmental history. Good sun exposure was defined as at least 15–30 min daily of exposure to the afternoon sun between 12.00 noon and 4 pm.

Physical examination included general examination and signs of rickets thus; bossing of the skull, craniotabes, widened wrists, bowed legs or knock knees, Harrison’s groove, spine deformities and beading of the ribs. Anthropometry measurements were taken including weight in kilogrammes and length in centimetres using an infantometer and stadiometer for children aged 2 years & below and older than 2 years respectively.

### Laboratory and radiological investigations

Five millilitres of blood was drawn for serum calcium, phosphorus, alkaline phosphatase and serum albumin. The biochemical tests were measured using Bayer Corporation Device® and ADIVA® analyzer at the Mulago Hospital Laboratory.

The calcium level of 8-10 mg/dl (2–2.5 mmol) was considered normal, phosphorus level: normal range infancy; 4.5–8.3 mg/dl (1.45–2.68 mmol), childhood; 3.7–5.6 mg/dl (1.19–1.8 mmol), alkaline phosphatase levels> 400 IU/dl was considered a raised level. The assays of 25 hydroxyvitamin D3 (25(OH) D3) were not done due to financial constraints.

**Fig. 1:**
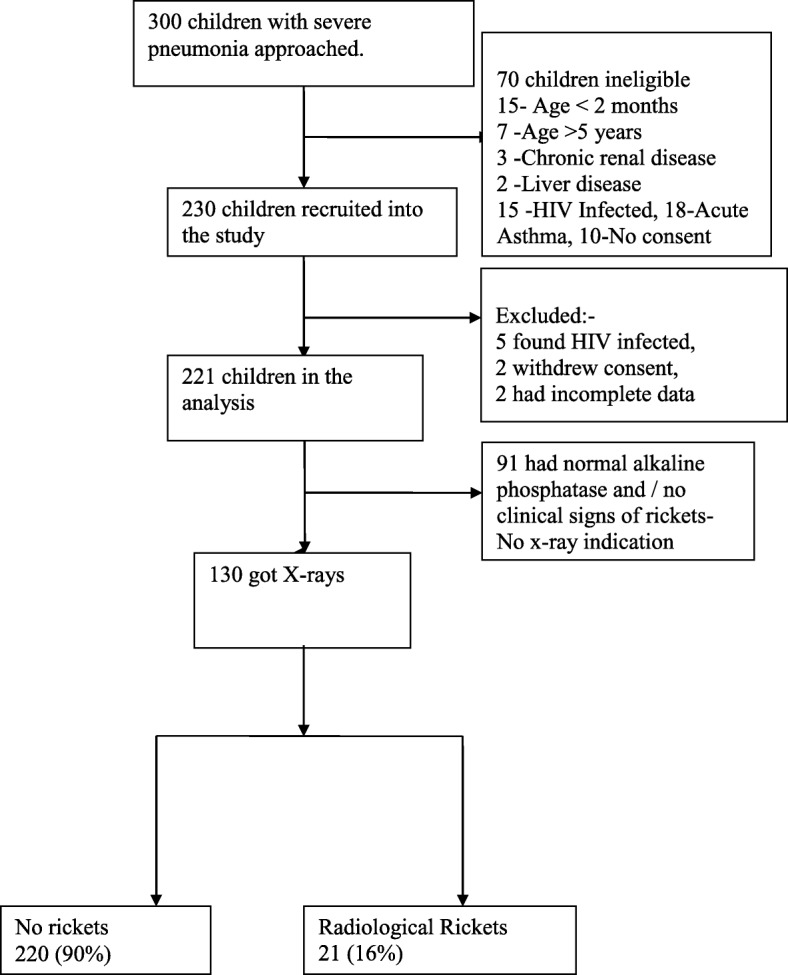
Showing the Study Profile of participant enrolment

All children with clinical signs and/or biochemical features of rickets (raised alkaline phosphatase) had a postero-anterior wrist x-ray done for radiological signs of rickets. Figure 1 shows the study profile. The radiological changes of rickets including fraying, widening and cupping of metaphysis were considered as rickets. The wrist radiographs of the patients were reported by a senior radiologist. Nutritional Rickets in this study was defined as the presence of any of the radiological changes of rickets on wrist X-ray.

All children identified with rickets were put on the stoss therapy. A dose of 150,000 IU for age less than 12 months and 300,000 IU of vitamin D for those older than 12 months was given. Children with low calcium were supplemented with oral calcium.

#### Data analysis

The study was powered at 80%, at an absolute error between the estimated and true value of 5%, with a 95% confidence interval. We made an assumption that the prevalence of rickets will be 17% as reported by Alan Smyth et al. in a study to detect radiological rickets in children with severe pneumonia in Zambia [7]. The sample size calculated was 217 children.

Statistical analysis was performed using the Stata 10 statistical package. The prevalence of rickets was calculated as the proportion of children with rickets among all those enrolled in the study. To determine the factors associated with rickets, categorical variables were compared between the two groups using the chi-square test, the means of continuous variables were compared using the Student’s t-test. Multivariable logistic regression was performed; all variables found to have a *P* value ≤0.2 at bivariate analysis were entered into the model. The height-for-age and weight-for-height *Z* scores were calculated from weights and heights using the Center for Diseases Control (CDC) standard charts. A *P*-value less than 0.05 was considered significant.

### Results

### Characteristics of participants

Table 1 shows the characteristics of the participants. The median age of the participants was 10 months (range 2–60), 84% of the participants were aged less than 24 months with majority being males (57.9%). Overall, 99% were born at term with favourable birth weights between 2.5–3.5 kg (67%). Sun exposure in this population was good, 80% of participants reported to have good sun exposure. Exclusive breastfeeding for at least six months was reported in 34.9% of the participants; with 24.9% and 10% reporting 6 and more than 6 months respectively of exclusive breastfeeding. Only 12% of the mothers were vegetarians.

**Table 1:** Socio-demographic characteristics of study participants

Characteristic	Frequency	Percentage (%)
Sex		
Male	128	57.9
Female	93	42.1
Age Group (months)		
2–5	49	22.3
6–23	135	61.3
24–60	36	16.4
Birth-order of child		
1st	50	22.6
2nd - 5th	160	72.4
> 5th	11	5.0
Birth Gestation age		
Term	218	98.6
Prematurity	3	1.4
Birth Weight (Kg)		
2.5–3.5	149	67.4
< 2.5	31	14.0
> 3.5	41	18.6
Previous admissions		
Yes	86	38.9
No	135	61.1
Breastfeeding		
Yes	141	63.8
No	80	36.2
Months of exclusive Breastfeeding		
< 6mths	144	65.1
6mths	53	24.0
> 6mths	24	10.9
Vegetarian Mothers		
Yes	26	11.8
No	195	88.2
Delay in milestones		
Yes	46	20.8
No	175	79.2
Exposure to the sun every day^a^		
Yes	177	80.1
No	44	19.9
NOK^b^ – Level of education		
None	20	9.1
Primary	92	41.6
Secondary/Tertiary	109	49.3

^a^Exposure to the sun was defined as Yes if the child was exposed to the sun at least 15- 30mins daily

^b^*NOK* – Next of Kin

### Prevalence of rickets

Twenty-one of the one hundred thirty children who had an x-ray done had radiological features of rickets. Therefore the prevalence of radiological rickets is 16% among children with severe pneumonia. The prevalence of nutritional rickets among the children enrolled is 9.5%.

However, 14.5% of the participants in this study had a raised Alkaline phosphatase. Only 10 of the 22 children with low calcium and 10 of the 35 children with low phosphorus had a raised ALP.

### Factors associated with rickets in severe pneumonia

Table 2 shows factors associated with rickets in children with severe Pneumonia at bivariate analysis. Poor sun exposure, low serum phosphorus, low serum calcium and high alkaline phosphatase were associated with rickets at bivariate analysis. However, at multivariate analysis, only a high alkaline phosphatase was independently associated with rickets (*p*-value < 0.001) as shown in Table 3. None of the clinical features was independently associated with rickets among children with severe pneumonia. The median age of the children with rickets in our study was 9 months, (range 2–24). Fifteen of 21 children with rickets were ages < 12 months and 75% of children with rickets were still breastfeeding. Only 10 of the 21 children with rickets had beading of the ribs detected while 18 reported delayed dentition. A third of the participants (32%) were wasted; however, wasting was not significantly associated with rickets. Hypocalcemia was uncommon in the participants at 10%. None of the participants were on any multivitamin supplements containing vitamin D.

**Table 2:** Factors associated with Rickets among children with severe Pneumonia

Variable	Rickets Status			
	Yes (%)	No (%)	OR (95% CI)	p-value
Sex				
Male	13(5.9)	115(52.0)	1	
Female	8 (3.6)	85(38.5)	0.83(0.33–2.10)	0.698
Age group				
< 24 months	19 (8.6)	165 (75.0)	1	
≥ 24 months	2 (0.9)	34 (15.5)	0.51 (0.11–2.30)	0.381
Birth Weight				
< 2.5 kg	3 (1.4)	28 (12.7)	1	
≥ 2.5 kg	18 (8.1)	172 (77.8)	0.98 (0.27–3.53)	0.971
No. of previous admissions				
None	14 (6.3)	121 (54.7)	1	
2 or more	7(3.2)	79 (35.8)	0.77 (0.30–1.98)	0.582
Breastfeeding				
Yes	16 (7.2)	125 (56.6)	1	
No	5(2.3)	75 (33.9)	0.52 (0.18–1.48)	0.221
Vegetarian				
Yes	3 (1.4)	23 (10.4)	1	
No	18 (8.1)	177(80.1)	0.78(0.21–2.86)	0.707
Delay in milestones				
Yes	7(3.2)	39 (17.6)	1	
No	14(6.3)	161(72.9)	0.48 (0.18–1.28)	0.144
Exposure to sun				
Yes	13 (5.9)	164(72.2)	1	
No	8 (3.6)	36 (16.3)	2.80 (1.08–7.26)	0.034
^a^WAZ score				
> −2 z score	13 (6.6)	120 (60.6)	1	
wasting				
≤ −2 z score	4 (2.0)	61 (30.8)	0.61 (0.19–1.94)	0.397
Any clinical Features of rickets				
No	14 (6.3)	152(68.8)	1	
Yes	7 (3.2)	48 (21.7)	1.58 (0.60–4.15)	0.350
Serum Ca				
Normal/high	14 (6.3)	185(83.7)	1	
Low	7(3.2)	15(6.8)	6.17(2.16–17.60)	0.001
Serum phosphorus				
Normal	13 (5.9)	172(78.2)	1	
Low	8 (3.6)	27 (12.3)	3.92(1.49–10.34)	0.006
Alkaline Phosphatase				
Normal/low	5(2.3)	184 (83.3)	36.8 (11.93–113.54)	0.000
High	16 (7.2)	16 (7.2)		

^a^*WAZ* –weight for age z score

**Table 3:** Multivariate Analysis: Factors independently associated with Rickets among Children with Pneumonia

Variable		P-value	Odds Ratio (CI)
Delay in Milestones	Yes versus No	0.341	0.53(0.14–1.96)
Exposure to the sun	Yes versus No	0.860	0.89 (0.24–3.28)
Serum calcium	Normal/high versus Low	0.610	1.49 (0.32–6.92)
Serum phosphorus	Normal/High versus Low	0.126	2.61 (0.76–8.92)
Alkaline Phosphatase	Normal/low versus High	0.000	32.95(10.54–102.93)

## Discussion

The prevalence of rickets in this study is quite low as compared to many other African studies carried out in a similar study population, with adequate sunshine exposure and similar socioeconomic status. The prevalence of rickets among children with pneumonia ranged from 17 to 80% in the various studies [3, 6, 12].

The discrepancy in the results may be due to the definition of rickets in this study as compared to the other African studies. Muhe et al. [12] in Ethiopia, defined rickets using both clinical and radiological features in children with pneumonia plausibly explaining the higher prevalence in their study. However, even with the just radiological definition of rickets they still found a high prevalence of rickets of 38% among the children with Pneumonia. A study in Zambia by Alan et al. [7] found a prevalence of 17% and all their rachitic cases were on the basis of osteopenia and not the typical features of rickets on the wrist x-ray, therefore the possible difference with our study. Another plausible explanation of the low prevalence of rickets in our study may be due to the fact that only about 60% of the study participants were x-rayed from our study algorithm. We could have missed a few radiological changes in those without an x-ray done, however, our assumption was that for radiological changes to occur, there should have been at least a clinical or biochemical change [13]. Although, some reports show that ALP may be normal in a few children with radiological changes, we believe only a negligible number of children may have been missed by our study algorithm. Therefore if the prevalence was calculated using only those with x-rays our prevalence would be 16%, very similar to a study done in Zambia. Another study in India [6] found a prevalence of rickets of 74% among those with severe pneumonia. The definition of rickets in their study was the finding of biochemical changes (raised alkaline phosphatase or low phosphorus or low calcium) with or without radiological evidence of rickets. They defined rickets as raised ALP above 200 U/L which may have caused over diagnosis of rickets. The use of alkaline phosphatase with a cut off value of 552 U/L has a high specificity for detecting nutritional rickets [14]. However the variation in assays from different laboratories makes it less reliable as each laboratory needs to establish its own cut –off value. In our study we used the radiological diagnosis of rickets to improve objectivity. In this study, elevated serum alkaline phosphatase was independently associated with rickets. Alkaline phosphatase is a sensitive marker that should be utilised to screen for rickets in our setting although it has a high sensitivity with a risk of over diagnosis of rickets. In resource limited settings where the laboratory assessment for vitamin D assays, calcium and phosphorus are not readily available, alkaline phosphatase will be a valuable test because it’s cheap and readily available. In this study very few children had rachitic clinical features, in a setting where assessment and investigations are dependent on clinical examination, many children with rickets may be missed during examination and only be diagnosed with advanced disease. Therefore we need to improve our index of suspicion and screen children with pneumonia for rickets even in settings with ample sunlight.

The prevalence of hypocalcaemia in this study was low and was not independently associated with rickets. Children with calcium deficiency rickets may have normal calcium due to the compensatory effects of parathyroid hormone to maintain the serum calcium however parathyroid hormone was not measured in this study. Studies in Nigeria [15–17] and South Africa [4, 8] have reported rickets as a result of calcium deficiency and not vitamin D deficiency in Africa because of abundance of the sun. Findings of calcium deficiency rickets in Africa were reported in older children aged 4 years and above, whose main diet comprised of foods high in phytates and oxalates [18] but the majority (76%) of children with rickets in this study were aged less than twenty-four months and were still breastfeeding thus calcium deficiency was less likely to be the cause of rickets. Breast milk contains calcium that is more readily absorbed for children than calcium in many other foods and has low vitamin D. Children from other African countries have also reported vitamin D deficiency rickets despite sun abundance [19, 20]. Unfortunately, in our study serum vitamin D levels were not measured due to cost constraints, vitamin D measurement could have differentiated between calcium deficiency and Vitamin D rickets.

Exposure to the sun was not significantly associated with rickets in this study and this could be because of the difficulty in estimating each child’s definite exposure like in other studies [12, 21]. Other factors that have been independently associated with rickets in other studies [6, 12, 21] like the duration of breastfeeding, family size, malnutrition and birth order were not significant in this study. This is possibly due to a better socioeconomic status of the participants with the majority of the mothers having a formal education.

This study had limitations, inability to assess 25 hydroxy Vitamin D due to financial constraints made it difficult to differentiate Vitamin D deficiency from calcium deficiency rickets. We were also unable to assess dietary intake of all the children and the breastfeeding mothers in order to determine the dietary intake of calcium and vitamin D. In addition, assessment of some of the variables like the history of sun exposure were very subjective and with recall bias.

## Conclusion

The prevalence of nutritional rickets is high among the children with severe pneumonia considering the ample sunshine in this study setting. Alkaline phosphatase is a good screening marker for rickets. Clinicians should actively assess children for rickets in this setting and screen for rickets in those children aged less than 24 months even without clinical features.

## Abbreviations

25(OH)D: 25 hydroxyvitamin D
ALP: Alkaline phosphatase
ALRI: Acute lower respiratory tract infection
WHO: World Health Organisation
